# Analyses of Long Non-Coding RNA and mRNA profiling using RNA sequencing in chicken testis with extreme sperm motility

**DOI:** 10.1038/s41598-017-08738-9

**Published:** 2017-08-22

**Authors:** Yifan Liu, Yanyan Sun, Yunlei Li, Hao Bai, Fuguang Xue, Songshan Xu, Hong Xu, Lei Shi, Ning Yang, Jilan Chen

**Affiliations:** 1 Key Laboratory of Animal Genetics Breeding and Reproduction (poultry), Ministry of Agriculture, Institute of Animal Science, Chinese Academy of Agricultural Sciences, Beijing, 100193 China; 2 China Agricultural University, Beijing, 100193 China; 3 Poultry Institute, Chinese Academy of Agricultural Sciences, Yangzhou, 225125 China

## Abstract

Sperm motility is the most important indicator in evaluating roosters’ fecundity. However, the genetic mechanisms underlying chicken sperm motility is not yet clear. Long non-coding RNA (lncRNA) play epigenetic roles in reproduction. In this study, RNA sequencing was employed to profile the testis transcriptome (lncRNA and mRNA) of six Beijing-you cocks divergent in sperm motility. In total, 2,597 lncRNAs were identified in the chicken testis, including 1,267 lincRNAs, 975 anti-sense lncRNAs, and 355 intronic lncRNAs. They shared similar features with previous studies. Of these lncRNAs, 124 were differentially expressed. Among 17,690 mRNAs detected in this study, 544 were differentially expressed, including a bunch of genes with known functions on sperm motility. GO annotation analysis revealed these genes were involved in ATP binding, cilium assembly, and oxidation-reduction processes. Integrating analysis of lncRNA and mRNA profiles predicted 10 lncRNA-gene pairs, including 8 co-regulated and 2 inversely-regulated pairs. To the best of our knowledge, this is the first genome-wide investigation of the lncRNAs in the chicken testis associated with sperm motility. Our results provided a catalog of chicken testis lncRNAs and genes worthy of further studies to understand their roles in cocks’ reproductive performance regulation.

## Introduction

Male’s reproductive efficiency play a key role in the economic success of livestock production. Defined as the proportion of forward-moving spermatozoa in an ejaculate, sperm motility is the most important indicator in evaluating roosters’ fecundity^1, 2^. A high proportion (>15%) of roosters with poor sperm motility was found in indigenous chicken breeds including Beijing-you (BJY) studied here^3^, severely impeding the genetic improvement. In view of the importance of sperm motility in males’ reproduction, increasing attention has focused on the genetic regulation of this trait. Sperm motility is high heritable^3, 4^, indicating the underlying genetic determinants. Better understanding of the related molecular regulation mechanisms may provide new insight and strategies to improve this trait. As the site of spermatogenesis and testosterone production, the testis play a central role in the male reproductive system. In mammals, genes expressed in the testis are associated with sperm motility, such as estrogen receptor (*ESR1* ^5^ and *ESR2* ^6^), CD9 molecule^7^, relaxin^8^, and retinol binding protein 4^9^.

Long non-coding RNA (lncRNA), one of the most high-expressed ncRNAs in animals, exerts critical roles through regulating gene expression in diverse biological processes including reproduction^10–12^. LncRNAs have been identified in the testis of human^13^, mouse^14^, and pig^15^, and play crucial roles in sperm development^16^. Knocking out of *Tsx*, a testis-specific lncRNA, causes an increase in apoptosis of pachytene spermatocytes in mice^17^. The expression profile of lncRNAs in chicken testis and functions associated with sperm motility remain unknown.

In the present study, a high-throughput RNA sequencing (RNA-seq) was employed to profile the testis transcriptome of six Beijing-you cocks divergent in sperm motility. The aims were to discover and characterize lncRNAs in chicken testis tissue and identify key genes, lncRNAs, and pathways that are associated with sperm motility. To the best of our knowledge, this is the first genome-wide investigation of the lncRNAs in the chicken testis associated with sperm motility. Our findings enable a better understanding of the underlying mechanisms implicated in cocks’ reproductive performance.

## Results

### Overview of sequencing and identification of lncRNA in chicken testis

In this study, six cDNA libraries were constructed using total testicular RNA from 3 Beijing-you cocks of high sperm motility (H1, H2, and H3) and 3 with low sperm motility (L1, L2, and L3). After quality control, approximately 9.3 gigabase (Gb) high-quality sequence data was remained for each sample. More than 73.1% of the total clean reads were mapped to the *Galgal 4.0*, and 61,369 assembled transcripts were produced. The detailed information of data quality and mapping statistics was presented in Supplementary Table S1.

**Figure 1:**
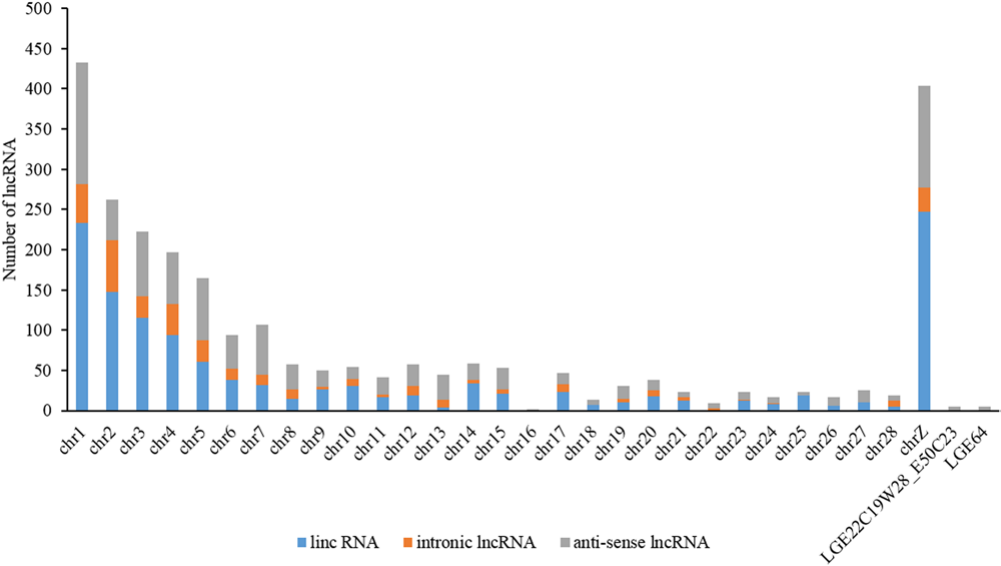
The distribution of testis lncRNA on chicken chromosome.

We developed a stringent filtering pipeline to identify reliable candidate lncRNAs from assembled transcripts (Supplementary Figure S1). Three different tools (CNCI, CPC and Pfam-scan) for calculating protein coding potency of transcripts were used in present study (Supplementary Figure S2). As a result, 2,597 candidate lncRNAs in 1,798 loci were captured, including 1,267 lincRNAs (48.8%), 975 anti-sense lncRNAs (37.5%), and 355 intronic lncRNAs (13.7%) (Supplementary Table S2). Of these lncRNAs, 517 (19.9%) were identified as known lncRNA by blasting against the known chicken lncRNAs in ALDB database (Supplementary Table S3). These lncRNAs distributed in all chromosomes. Notably, 433 (16.7%) and 404 (15.6%) lncRNAs were located in the chromosome 1 and Z, respectively (Fig. 1).

### Genomic feature of lncRNAs

**Figure 2:**
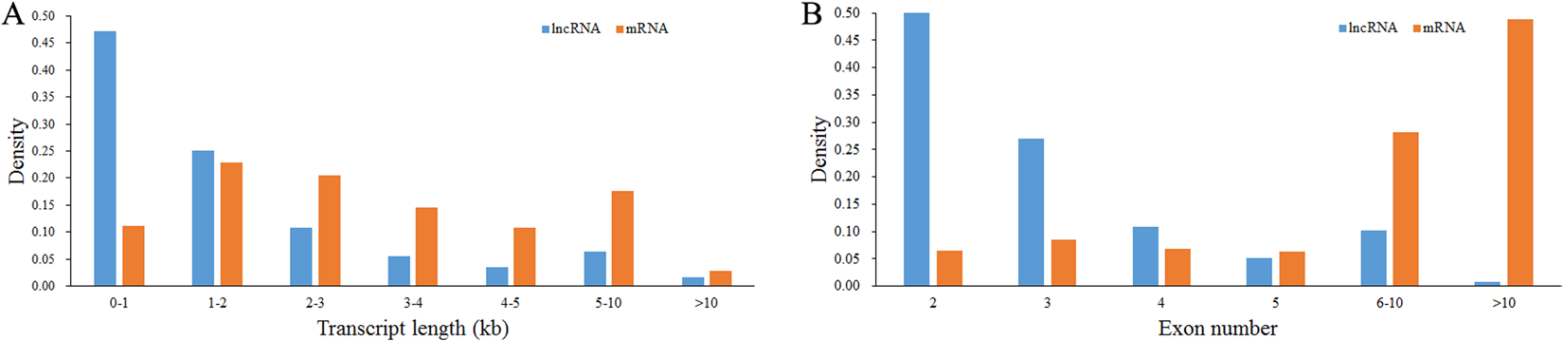
Genomic features of predicted lncRNAs. (**A**) Length distribution of lncRNAs and mRNAs. (**B**) Exon number distribution of lncRNAs and mRNAs.

Comparing to the 17,690 protein-coding transcripts identified in our study, we found chicken lncRNAs (1,918 bp on average) were significantly shorter than the mRNAs (3,485 bp on average). As shown in Fig. 2A, 72.2% of lncRNAs and 57.8% of mRNAs have a length of 0–2,000 bp and 1,000–4,000 bp, respectively. Moreover, the number of exons of lncRNAs (2.97 on average) was less than that of mRNAs (12.18 on average). The majority of lncRNAs (78.2%) have 3 or less exons, while 76.9% of mRNAs have 5 or more (Fig. 2B). The lncRNAs in the testis were shorter than those in the skeletal muscle (2,941 bp on average), while the number of exon was similar^18^. After quantified the expression level of transcripts by Stringtie, the overall expression level of lncRNAs was lower than mRNAs (Supplementary Figure S3).

The sequence conservation test showed that only 9 chicken lncRNAs were overlapped with 29 human lncRNAs, and 9 overlapped with 14 mouse lncRNAs (Supplementary Table S4). Additionally, five of the lncRNAs overlapped both with human and mouse.

We found that 1,525 lncRNA loci were transcribed near (<100 kb) their protein-coding neighbors, 1,895 in total (Supplementary Table S5). Of these neighbors, 1,611 were assigned to 15 biological processes in GO enrichment (Enriched genes > 10, Supplementary Table S6), mainly referred to signal transduction and transcription regulation.

### Differential expression mRNAs and lncRNAs

**Figure 3:**
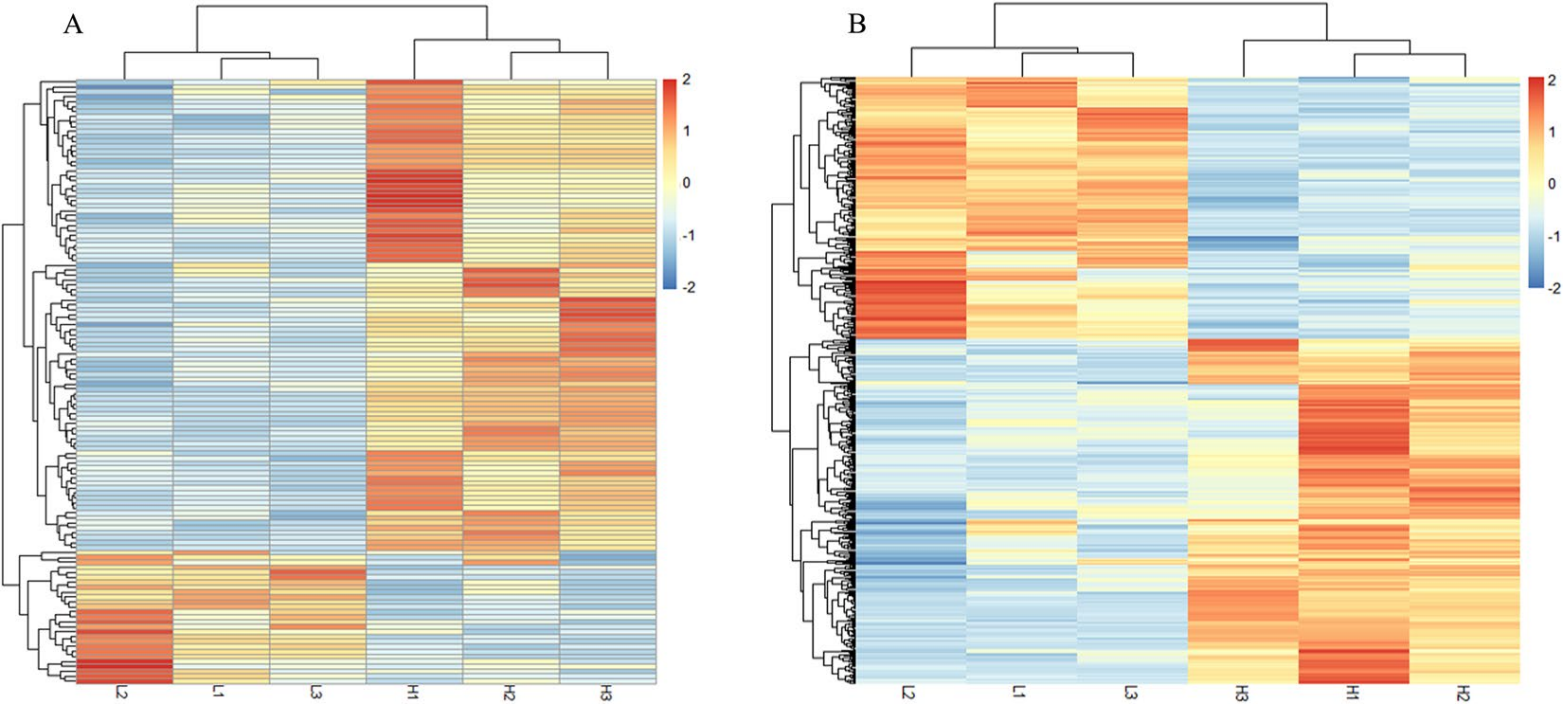
Heat maps of the distinguishable expression profiles in chicken testis between low and high sperm motility groups. (**A**) Hierarchical clustering of the differentially expressed lncRNA. (**B**) Hierarchical clustering of the differentially expressed mRNAs.

A transcript-level expression analysis was conducted to detect the differentially expressed lncRNAs and mRNAs between high and low sperm motility groups using ballgown R package. Taking *P*-value < 0.05 and |foldchange| > 1.5 as the cutoff, 29 lncRNAs and 235 mRNAs were found to be up-regulated in the low sperm motility group, while 95 lncRNAs and 309 mRNAs were down-regulated (Supplementary Tables S7 and S8). The heatmaps displayed differentially expressed lncRNA (Fig. 3A) and mRNAs (Fig. 3B).

**Figure 4:**
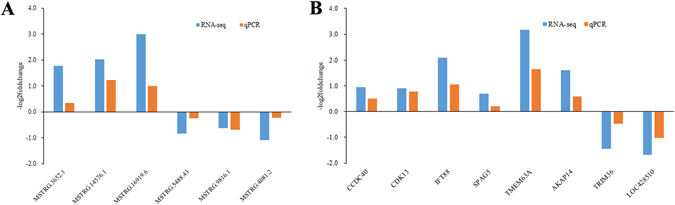
Illustrating of qPCR confirmation for RNA-seq. (**A**) Validation of 6 selected differential lncRNAs; (**B**) Validation of 8 selected differential mRNAs.

The differentially expressed lncRNAs obtained in our study included 56 lincRNAs, 13 intronic lncRNA, and 55 anti-sense lncRNAs. With the potential target gene predicting in *cis*, 10 target genes related to 10 lncRNAs were identified. As shown in Table 1, eight of the lncRNA-gene pairs were regulated in the same direction (co-regulated) and two pairs in the opposite direction (inversely-regulated). We also observed higher correlation coefficients between the lncRNAs and their neighboring genes than the mean correlation coefficient (0.0270) between random gene pairs. In addition, the expression of lncRNA MSTRG.3652 and MSTRG.4081, and their potential target gene *CDK13* and *LOC428510* in low and high sperm motility groups were further validated by performing qPCR (Fig. 4).

**Table 1:** Differentially expressed lncRNA-gene pairs between low and high sperm motility groups.

Gene symbol	Foldchange (L/H)	LncRNA ID	Foldchange (L/H)	Regulation type	Correlation coefficient	*P*-value
*AHI1*	−2.14389	MSTRG.5628	−3.1614	Co-regulated	0.4323	0.0249
*AK5*	−1.52226	MSTRG.10220	−1.65421	Co-regulated	0.8234	0.0035
*BIRC6*	2.007408	MSTRG.5281	−1.60718	Inversely-regulated	−0.6532	0.0312
*CDK13*	−1.86264	MSTRG.3652	−1.69682	Co-regulated	0.5735	0.0232
*KIRREL3*	2.14843	MSTRG.15407	2.33021	Co-regulated	0.6057	0.0146
*LOC100858942*	−1.47349	MSTRG.17773	−1.80294	Co-regulated	0.7584	0.0345
*LOC101749614*	−2.32507	MSTRG.17846	−2.8401	Co-regulated	0.5039	0.0213
*LOC426324*	−1.91267	MSTRG.11244	−2.0126	Co-regulated	0.3554	0.0198
*LOC428510*	3.190001	MSTRG.4081	2.126971	Co-regulated	0.6132	0.0376
*TTLL2*	−2.33525	MSTRG.5488	1.79202	Inversely-regulated	−0.6451	0.0278

Pearson correlation coefficient between expression of lncRNAs and their target genes was calculated.

### Function enrichment related to sperm motility

**Figure 5:**
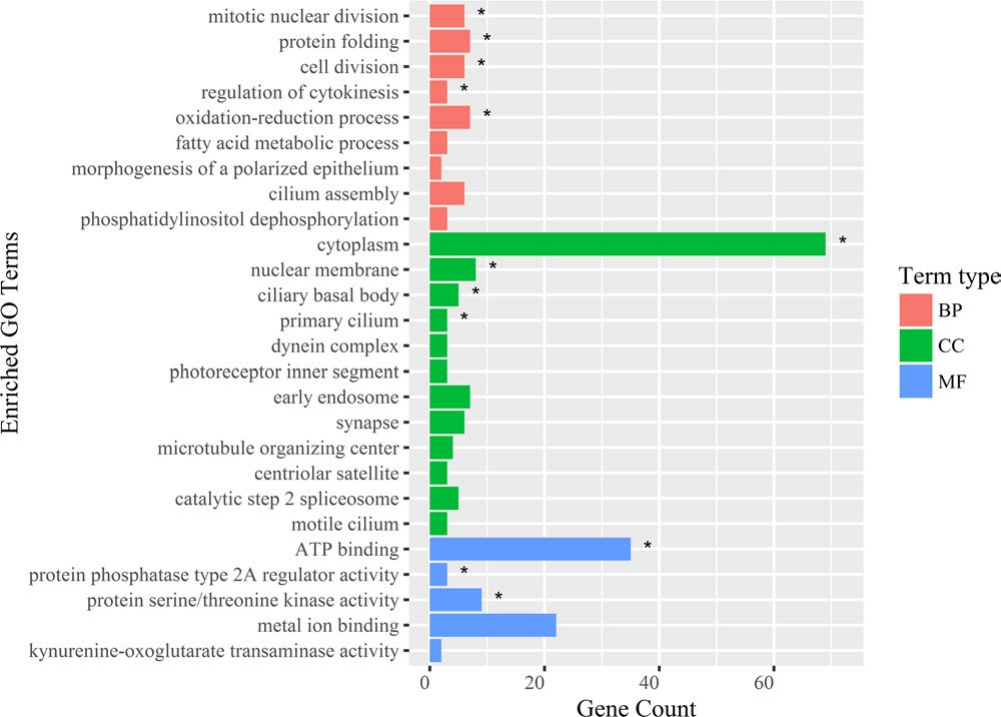
GO analysis of differentially expressed genes between low and high sperm motility groups. BP: biological process; CC: cellular component; MF: molecular function. The marked “*” means significantly enriched GO terms.

The 544 differentially expressed genes with functional annotation information were assigned to 1,236 GO terms. There were 5, 4, and 3 GO terms significantly enriched for biological process, cellular components, and molecular function, respectively (*P*-value < 0.05) (Fig. 5). The differential genes were mainly involved in cilium assembly, cell division, ATP-binding, and oxidation-reduction process. It was noted that 14 GO terms including cilium assemble with *P*-value ranged between 0.05 and 0.10 was enriched in our study. Furthermore, the enriched GO terms associated with cell division, ion-binding were up-regulated, while the cilium related GO terms were more often associated with down-regulated genes (Supplementary Table S9).

### Quantitative real-time PCR (qPCR) validation

To validate the RNA-seq results, 8 differentially expressed mRNAs (*CCDC40*, *CDK13*, *IFT88*, *SPAG5*, *TMEM63A*, *AKAP14*, *TRIM36*, and *LOC428510*) and 6 differentially expressed lncRNAs (MSTRG.3652.1, MSTRG.14576.1, MSTRG.16919.6, MSTRG.5488.43, MSTRG.4081.2 and MSTRG.9816.1) were selected for qPCR. As shown in Fig. 4, the relative foldchange in qPCR were consistent with RNA-seq results, suggesting that the transcript identification and abundance estimation were highly reliable.

## Discussion

Sperm motility is an important economic trait in poultry production. However, the mechanism underlying this trait is not clear yet. LncRNAs have received much attention in the past decade, and are found to play vital roles in male fertility^16^. In the present study, Illumina high-throughput sequencing was performed to provide an extensive lncRNA and gene expression profile in the chicken testis with high and low sperm motility. As a result, a total of 2,597 lncRNAs, 544 differentially expressed mRNAs, and 124 differentially expressed lncRNAs were obtained from two groups.

LncRNAs shared many common characteristics among species, including low expression, shorter transcript length, and fewer exons as compared to known protein coding transcripts^19, 20^. These features were also observed in this study, indicating that the lncRNAs identified here were reliable. Consistent with previous reports^14, 21^, we found that only 20% of lncRNAs were mapped with reported chicken lncRNAs from ALDB database, suggesting that lncRNAs in chicken testis tended to be highly tissue-specific. There were 433 (16.7%) and 404 (15.6%) of lncRNAs located on chromosome 1 and Z. Considering the length of chromosome 1 is 2-fold of chromosome Z, the high density of lncRNAs on chromosome Z suggests their roles in regulation of fertility. In addition, the function annotation of lncRNA neighboring genes indicates were preferentially located in the vicinity of protein-coding genes that are related to signal transduction and transcription regulation, which was similar with previous studies^22, 23^.

Analyzing conservation of lncRNAs across species might help understand lncRNA evolution and function^24^. In this study, less than 1% chicken lncRNA showed significant sequence similarities with human and mouse lncRNAs. It was also reported that less than 6% of zebrafish lincRNAs owned detectable sequence conservation with human or mouse lincRNAs^25^. These results indicate that lncRNAs is unlikely high conserved between species, especially in mammals and non-mammals.

Most evidence suggests that the expression of lncRNAs can regulate and have high correlations with expression of neighboring mRNAs^23, 26^. In the present study, the protein neighbors of 124 differentially expressed lncRNAs were used to predict their potential roles in regulation of sperm motility. Combing with differentially expressed genes, 10 pairs of lncRNAs and their target genes were both differentially expressed, and 2 of them show their potential in sperm motility regulation. LncRNA MSTRG.3652 was predicted to act on the target gene *CDK13*, a member of cyclin-dependent serine/threonine protein kinase (CDK) family. The CDK family is well known for their essential roles as master switches in cell cycle control^27^. Furthermore, CDKs have functions in germ cell development by regulating mitotic and meiotic divisions^28^. The knockdown of *CDK4*, another member of CDKs, induced infertile in mice^29^. In this study, the low expression of MSTRG.3652 and *CDK13* might contribute to the low sperm motility in chicken. *LOC428510* is predicted to be a target gene of lncRNA MSTRG.4081, and they were both differentially expressed here. *LOC428510* encodes a protein whose structure is similar to dynein heavy chain 5 (*DNAH5*). DNAH5 is known as an important motor protein of sperm movement^30^. Mutation of *DNAH5* gene may cause the sperm immobility^31^. In addition, the co-regulation of LOC428510 and lncRNA MSTRG.408 was further confirmed by qPCR. These findings suggested that MSTRG.3652, MSTRG.4081, *CDK13*, and *LOC428510* may be involved in the regulation of sperm motility in the roosters.

It is well established that sperm motility is highly dependent on several metabolic pathways and regulatory mechanisms, including sperm protein phosphorylation^32, 33^, sperm flagellar assembly^34^, and stress response^35^. ATP binding and serine/threonine kinases activity are two essential elements for phosphorylation which contributes to proper functioning of sperm proteins^30^. In this study, ATP binding and serine/threonine kinases activity were both enriched. The shared genes in these two processes including *CAMK4*, *SGK3*, *RPS6KA2*, *RPS6KC1*, *PRKAA1*, and *NEK6* were down-regulated in the low sperm motility group. Sharing similar structure with cilium, flagellum is important for sperm movement. In consistent with Zhuang’s work^36^, four GO terms associated with cilium were identified here. Another enriched process is oxidation-reduction. The balance of production of reactive oxygen species (ROS) and oxidation-reduction responses play essential roles in maintaining normal mechanisms of cellular signaling^37^. Increased ROS levels have been correlated with decreased sperm motility^38^. The oxidation-reduction process may be a key mechanism of sperm motility regulation. In this study, some genes with known functions related to sperm motility in mammals were also found to be differentially expressed, including *CCDC40* ^39, 40^, *AKAP14* ^41^, and *SPAG5* ^42^, indicating that these genes may be also important in poultry.

In conclusion, transcriptome sequencing was first used in this study to generate the expression profile of lncRNAs and mRNAs in chicken testis, and a bunch of differentially expressed lncRNAs and genes associated with sperm motility was obtained. Function enrichment analysis revealed that differentially expressed genes were involved in sperm protein phosphorylation, sperm flagellar assembly, and stress response, and shed light on their potential regulation roles of chicken sperm motility. The lncRNAs identified in chicken testis share similar features with previous studies, also show special characteristics such as high density on chromosome Z. LncRNA MSTRG.3652 and MSTRG.4081 may play roles in regulating the sperm motility by governing their potential target genes *CDK13* and *LOC428510*, respectively.

## Materials and Methods

### Ethics statement

All of the procedures involving animals were approved by the Animal Care and Use Committee at Institute of Animal Science, Chinese Academy of Agricultural Sciences (IAS-CAAS), where the experiment was conducted. All of the experiments were performed in accordance with the relevant guidelines and regulations set by Ministry of Agriculture of the People’s Republic of China.

### Sampling

All animals used in the present study were from a Beijing-you chicken pure line kept on the experimental farm of IAS-CAAS, Beijing, China. To collect cocks divergent in sperm motility for sequencing, sperm motility estimation was performed 10 times, at a 2-day interval, in 396 Beijing-you male breeders since 40 wk of age. Sperm motility was estimated by microscopic observation (×400 magnification)^43^, expressed as the number of motile spermatozoa with moderate to rapid progressive movement of 10. Based on the averaged sperm motility, 15 highest and 15 lowest phenotypes were selected at 45 wk of age to be used as semen donor to inseminate the females. The percentage of fertile egg of each rooster (fertility rate) was calculated after incubation to further verify the individual fertility capacity. At the end, three birds with high sperm motility and fertility rate, and 3 birds with low sperm motility and fertility rate were selected for RNA-seq. Threshold values for the high sperm motility group were sperm motility ≥7 and fertility rate ≥70%, and those for the low sperm motility group were sperm motility ≤3 and fertility rate ≤30%. The sperm motility and fertility rate of these cocks were shown in Table 2.

**Table 2:** Sperm motility and fertility rate of six cocks involved in RNA-seq.

	High sperm motility group				Low sperm motility group			
Sample	H1	H2	H3	Means	L1	L2	L3	Means
Sperm motility	7.05	7.40	8.14	7.53	0.78	2.30	2.41	1.83
Fertility rate (%)	72.3	89.1	74.4	78.6	0.1	10.8	25.7	12.1

At 50 wk of age, all 6 chickens were euthanized for tissue sampling. Testis samples were dissected, frozen in liquid nitrogen temporarily, and stored at −80 °C until further manipulation.

### Total RNA extraction

Total RNA was extracted from each individual testis sample using TRIzol reagent (Invitrogen, USA), following the manufacturer’s instructions. The concentration and integrity of RNA was estimated using the NanoDrop 2000 (Thermo, USA) and Agilent 2100 Bioanalyzer (Agilent Technologies, CA, USA), respectively.

### Library preparation for lncRNA sequencing

TruSeq Stranded Total RNA with Ribo-Zero Gold kit (Illumina, CA, USA) was used to generate strand-specific RNA-seq libraries following the manufacturer’s recommendations. In brief, Ribosomal RNA was removed from total of 3 μg RNA. After RNA fragmentation, double-stranded cDNA was synthesized replacing dTTPs with dUTPs in the reaction buffer used in second strand cDNA synthesis. The resulting double-stranded cDNA was ligated to adaptors after being end-repaired and A-tailed. Single strand cDNA was then obtained using USER Enzyme. PCR amplification was performed to enrich cDNA libraries. Finally, PCR products were purified and library quality was assessed on the Agilent Bioanalyzer 2100 system.

Sequencing was performed on an Illumina Hiseq 2500 instrument using the TruSeq PE Cluster Kit v3-cBot-HS (Illumina, CA, USA) to generate 150 bp paired-end reads.

### Quality control, mapping, and assembly of transcriptomic data

Quality control and reads statistics were determined by FastQC (0.11.2)^44^. Discarding the reads containing adapter or ploy-N and other low quality ones, the remaining clean reads were aligned to the reference chicken genome (*Galgal 4.0*) using Hisat (2.0.1)^45^. Mapped transcripts were assembled individually with Stringtie (1.2.4)^46^. Refgene annotation was supplied to guide assembly process. Transcripts from all samples were then merged together with Stringtie merge mode to build a consensus set of transcripts across samples.

### Identification of lncRNAs and their nearest neighbor genes

To reduce the false positive rates, assembled transcripts was put into following steps to gain candidate lncRNAs: (1) Transcripts with one exon and shorter than 200 bp were removed; (2) The reads coverage of transcript was calculated using Stringtie (1.2.4), and those with reads coverage less than 3 were eliminated; (3) Transcripts belong to coding-genes, pseudogenes, pre-microRNA, tRNA, rRNA, and snoRNA were removed; (4) Protein coding potency of transcripts were calculated by three software: Coding-Non-Coding-Index (CNCI)^47^ (score < 0), Coding Potential Calculator (CPC)^48^ (score < 0), and Pfam-scan^49^ (E-value < 0.001). Transcripts identified with coding potential in any of the three tools were filtered out.

Transcripts that passed all the filters mentioned above were considered as candidate lncRNA and were then blasted to chicken lncRNAs in the ALDB v1.0 database^50^. Refgene annotation was used to search the nearest neighboring genes of lncRNAs, and 100 kb was set as the threshold.

### Homology analysis with lncRNAs in human and mouse

Human and mouse lncRNAs were downloaded from GENCODE database^51^. Blastn method was used to search for homology between candidate lncRNAs identified in the GENCODE database, considering a 1 × 10^−6^ E-value threshold.

### Target genes prediction

Differentially expressed lncRNAs were selected for target prediction. The *cis* role of lncRNAs was their acting on neighboring target genes^52, 53^. In order to reduce false positives, the differentially expressed genes located within the 100 kb distance of the differentially expressed lncRNAs were selected as potential target genes. The Pearson correlation test was used to calculate the correlation coefficients between lncRNAs and their potential target genes, and that between the random gene pairs.

### Differential expression and function enrichment analysis

The quantification of lncRNAs and mRNAs in each sample was calculated by Stringtie. Differentially expressed mRNAs and lncRNAs between low and high sperm motility groups was analyzed using the ballgown (2.6.0) R package^54^. *P*-value < 0.05 and |fold-change| > 1.5 were considered as significance threshold. GO enrichment analysis of differentially expressed mRNAs and lncRNAs were performed using the DAVID (6.8) online tool (https://david.ncifcrf.gov/)^55^.

### qPCR validation

RNA samples from the testis of 6 individuals used for the RNA-seq were analyzed by qPCR. qPCR was performed on an ABI 7500 Real time PCR system (Applied Biosystem, USA) using KAPA SYBR Fast universal qPCR kit (Kapa Biosystems, USA). Specific primers of genes and lncRNAs as shown in Supplementary Table S10 were designed using the Primer Premier 5 and confirmed by Oligo 6.0. Using *GAPDH* as a reference, relative-expression levels of genes and lncRNAs were quantified using 2^(−ΔΔCt)^ methods^56^.

## Electronic supplementary material

Table S1

Table S2

Table S3

Table S4

Table S5

Table S6

Table S7

Table S8

Table S9

Table S10

Supplementary information
