# Supplementary material for: Analyses of Long Non-Coding RNA and mRNA profiling using RNA sequencing in chicken testis with extreme sperm motility
Source: Sci Rep. 2017 Aug 22;7:9055. doi: 10.1038/s41598-017-08738-9 (PMC5567338; doi:10.1038/s41598-017-08738-9)
Supplement: Supplementary file 11 — Supplementary information [file 41598_2017_8738_MOESM11_ESM.doc]

Supplementary Information

**Analyses of Long Non-Coding RNA and mRNA profiling using RNA sequencing in chicken testis with extreme sperm motility**

Yifan Liu^1,2,3§^, Yanyan Sun^1§^, Yunlei Li^1^, Hao Bai^1^, Fuguang Xue^1^, Songshan Xu^1^, Hong Xu^1^, Lei Shi^1^, Ning Yang^2^ and Jilan Chen^1^*

^1^Key Laboratory of Animal Genetics Breeding and Reproduction (poultry), Ministry of Agriculture, Institute of Animal Science, Chinese Academy of Agricultural Sciences, Beijing 100193, China.

^2^China Agricultural University, Beijing 100193, China.

^3^Institute of Poultry Science, Chinese Academy of Agricultural Sciences, Yangzhou 225125, China.

^§^These authors contributed equally to this work

Correspondence and requests for materials should be addressed to J.C. (email: chen.jilan@163.com)

**Figure S1 The pipeline of lncRNAs identification.**

**Figure S2 Venn graph showing number of identified lncRNAs from an intersection of CNCI, CPC and Pfam-scan results.**

**Figure S3 Box plots showing the expression feature of lncRNAs and mRNAs.**

**Table S1 Output statistics and annotation information of the sequencing reads for each sample.**

**Table S2 Details of identified lncRNAs in chicken testis.**

**Table S3 Identified lncRNAs included in chicken ALDB database.**

**Table S4 Identified lncRNAs with sequence similarities with human or mouse.**

**Table S5 Nearest neighboring genes of identified lncRNAs in chicken testis.**

**Table S6 GO analysis of the nearest neighboring protein-coding genes of lncRNAs.**

**Table S7 Differentially expressed lncRNAs identified in chicken testis between low and high sperm motility groups.** Foldchange = Expresstion(low group)/Expression(high group).

**Table S8 Differentially expressed mRNAs identified in chicken testis between low and high sperm motility groups.** Foldchange = Expresstion(low group)/Expression(high group).

**Table S9 GO analysis of up-regulated and down-regualted genes.**

**Table S10 Primers of lncRNAs and mRNAs used in qPCR.**


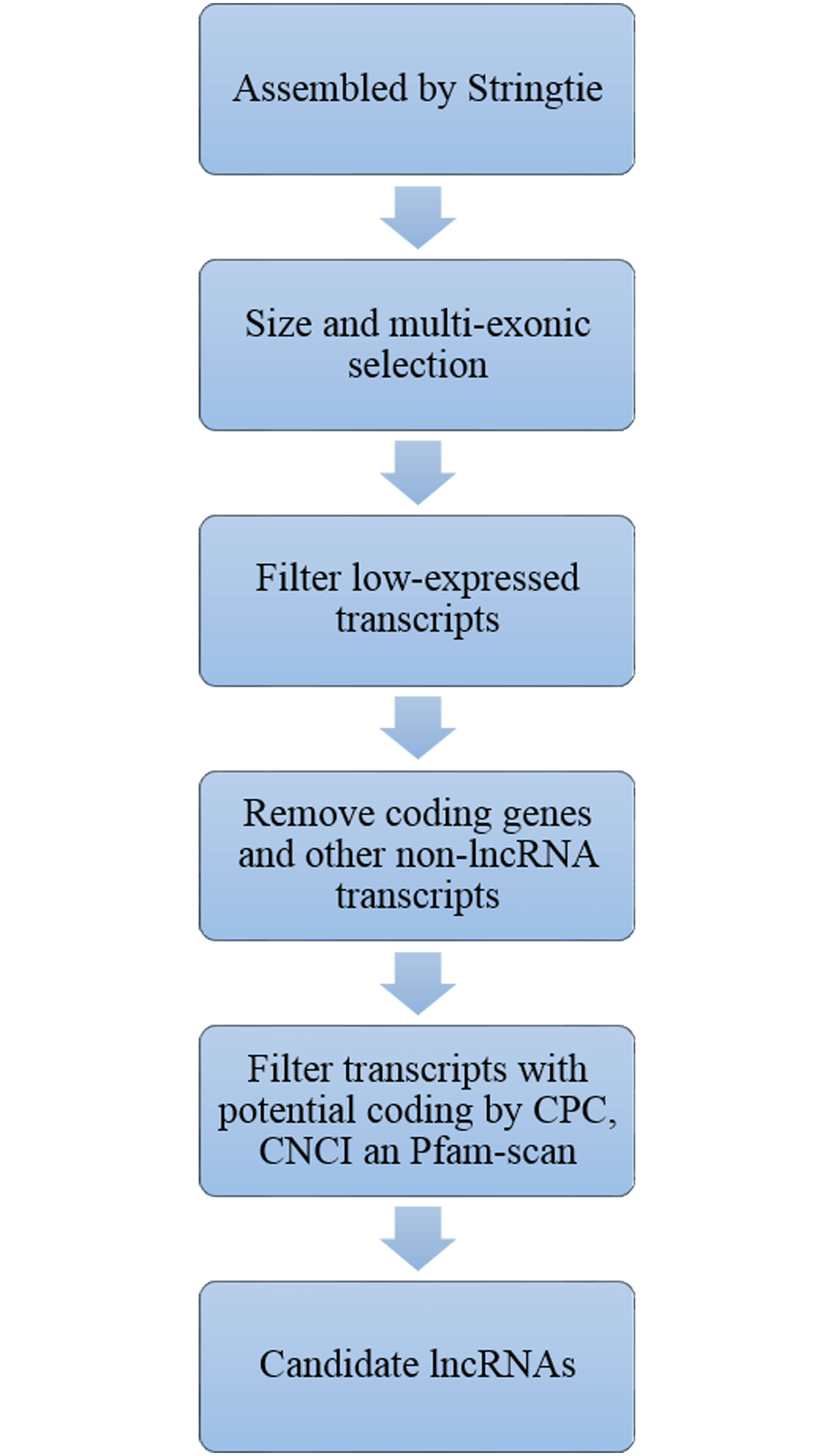


**Figure S1 The pipeline of lncRNAs identification.**

**
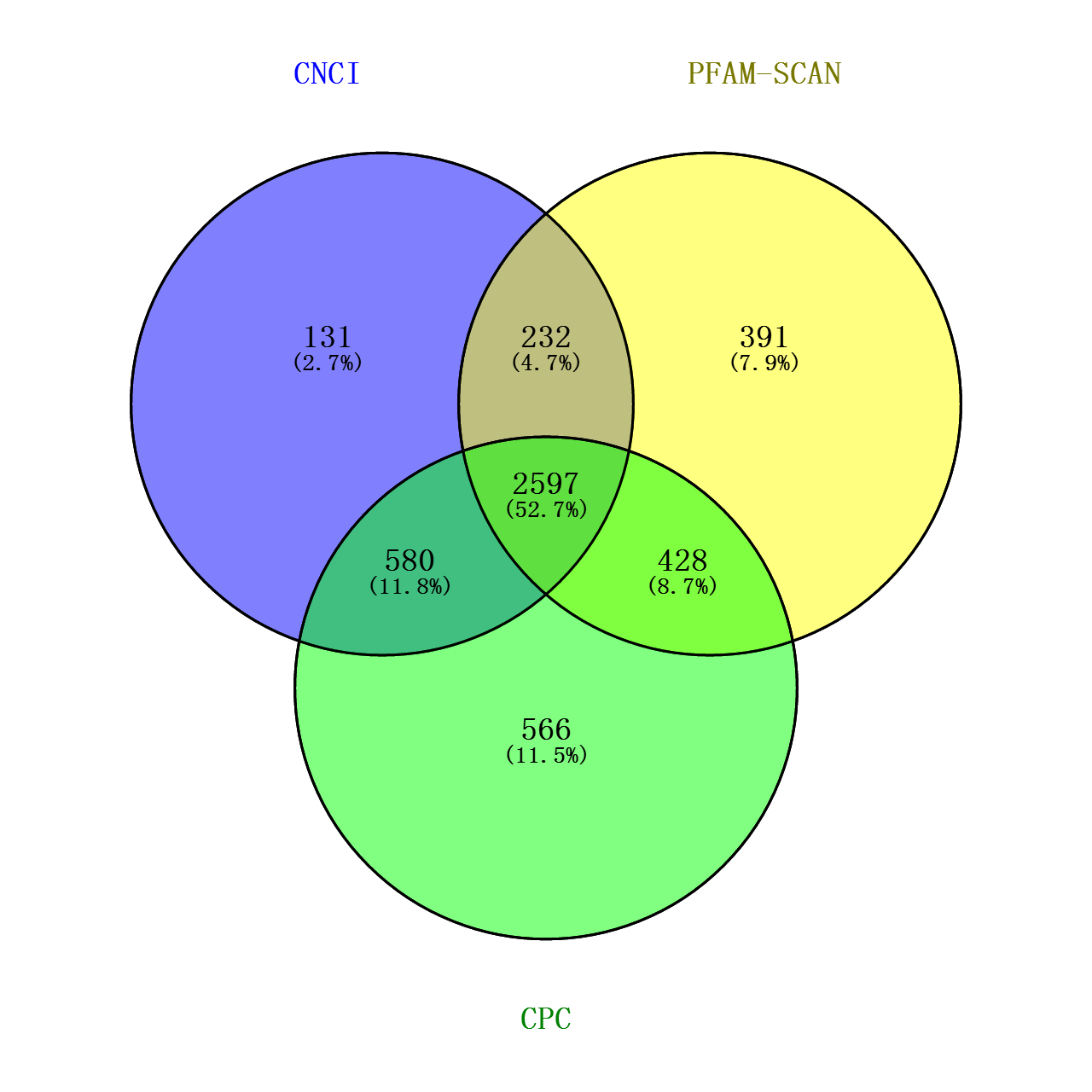
**

**Figure S2 Venn graph showing number of identified lncRNAs from an intersection of CNCI, CPC and Pfam-scan results.**


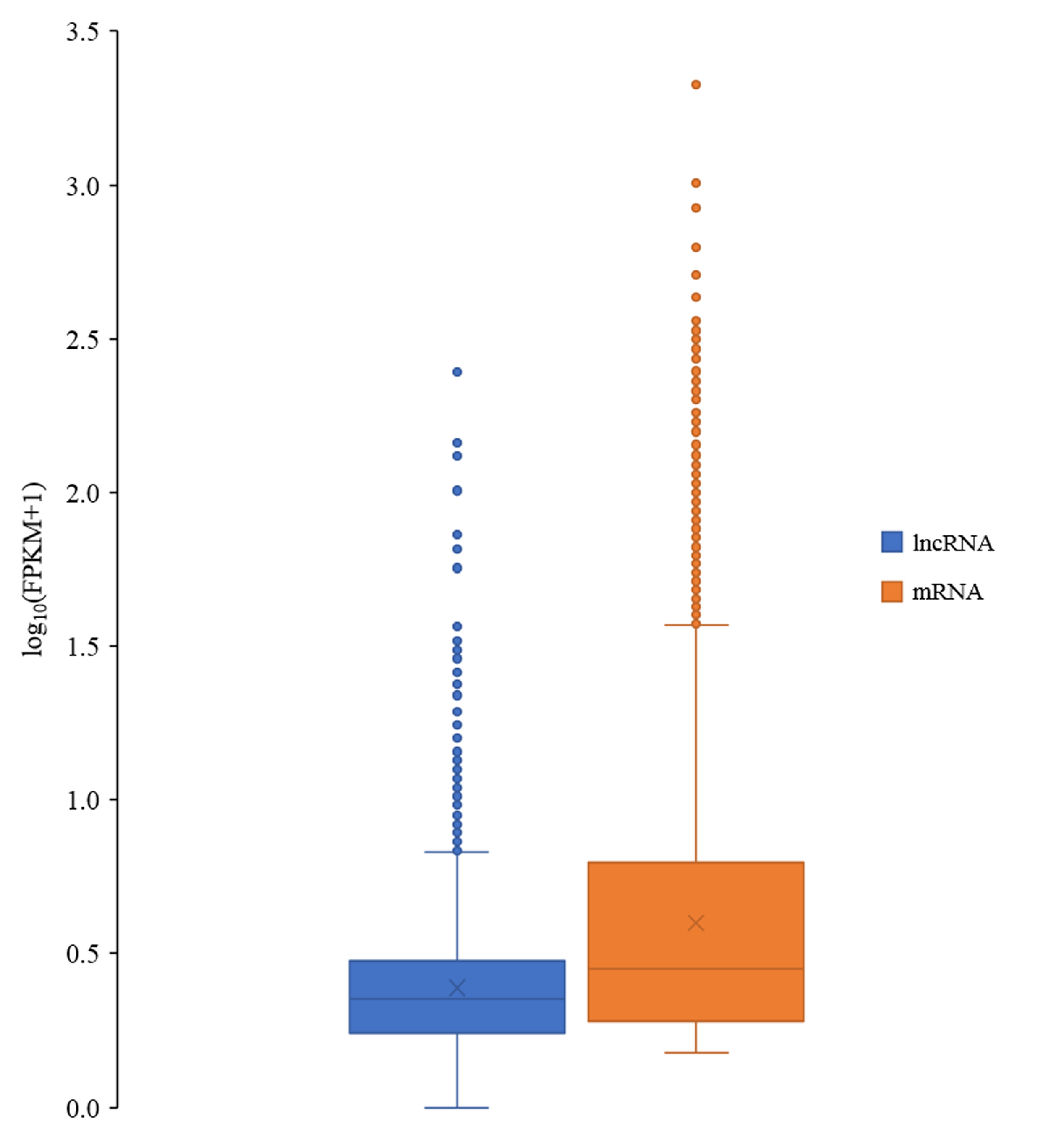


**Figure S3 Box plots showing the expression feature of lncRNAs and mRNAs.**
